# Mining a Sea of Data: Deducing the Environmental Controls of Ocean Chlorophyll

**DOI:** 10.1371/journal.pone.0003836

**Published:** 2008-11-27

**Authors:** Andrew J. Irwin, Zoe V. Finkel

**Affiliations:** 1 Department of Mathematics & Computer Science, Mount Allison University, Sackville, New Brunswick, Canada; 2 Environmental Science Program, Mount Allison University, Sackville, New Brunswick, Canada; Monterey Bay Aquarium Research Institute, United States of America

## Abstract

Chlorophyll biomass in the surface ocean is regulated by a complex interaction of physiological, oceanographic, and ecological factors and in turn regulates the rates of primary production and export of organic carbon to the deep ocean. Mechanistic models of phytoplankton responses to climate change require the parameterization of many processes of which we have limited knowledge. We develop a statistical approach to estimate the response of remote-sensed ocean chlorophyll to a variety of physical and chemical variables. Irradiance over the mixed layer depth, surface nitrate, sea-surface temperature, and latitude and longitude together can predict 83% of the variation in log chlorophyll in the North Atlantic. Light and nitrate regulate biomass through an empirically determined minimum function explaining nearly 50% of the variation in log chlorophyll by themselves and confirming that either light or macronutrients are often limiting and that much of the variation in chlorophyll concentration is determined by bottom-up mechanisms. Assuming the dynamics of the future ocean are governed by the same processes at work today, we should be able to apply these response functions to future climate change scenarios, with changes in temperature, nutrient distributions, irradiance, and ocean physics.

## Introduction

The ocean is one of the most important reservoirs of inorganic carbon and its ability to act as a long-term sink for CO_2_ is affected by phytoplankton through the flux of photosynthetically fixed carbon from the surface into the deep ocean, termed the biological pump. The standing stock of phytoplankton biomass is a primary determinant of the rates of primary production and export of carbon out of the surface ocean [1]. There is accumulating evidence that phytoplankton biomass and community composition are changing in response to climate change [2]–[5]. Models often have difficulty in accurately predicting changes in chlorophyll from physical or chemical parameters beyond small spatial and temporal scales due to the complex web of interacting processes that can affect the standing-stock [6]. Predicting how phytoplankton chlorophyll will respond to changes in climate, including temperature, nutrient availability, and ocean circulation will improve predictions of how climate change will alter the ocean's capacity to act as a carbon sink.

There are many approaches to describing the response of phytoplankton standing stock and the biological pump to climate change. The simplest is to provide upper and lower bounds on the magnitude of the biological pump by comparing an abiotic ocean with no phytoplankton to a super-biotic ocean in which all upwelled nutrient is incorporated into organic matter through phytoplankton photosynthesis [7], [8]. These approaches cannot predict how phytoplankton biomass and community structure will acclimate or adapt to particular environmental change scenarios. Alternatively, physiologically detailed mechanistic models incorporate the growth response of several biogeochemically defined groups of phytoplankton to the availability of light and several different potentially limiting nutrients balanced by loss terms such as sinking and grazing by different classes of predators [9]–[12]. These physiologically mechanistic models can simulate relatively rapid changes in phytoplankton growth rate, community composition and chlorophyll biomass. The quality of the output from these models predictions is proportional to current knowledge of the response of phytoplankton and the rest of the marine food web to the appropriate environmental variables. Even the most complex of these classes of models must make critical approximations, often greatly simplifying the physiological response of phytoplankton to limiting resources, viral and parasitic loss, competitive interactions within trophic levels, and neglecting many of the higher trophic levels and food web interactions entirely. Physiological responses of phytoplankton to light and nutrient availability are complex, often varying significantly between species and even ecotypes. Effects on biomass due to grazing, parasitism, and competitive interactions are poorly constrained due to limitations in both data and mechanistic understanding. Data essential to these models are also sometimes incomplete, for example the distribution of iron input and the proportion that is bio-available is still fairly uncertain in many areas of the ocean [13]. Ongoing work on all these fronts yields a continually evolving view of phytoplankton and their interaction with the marine environment.

Historically, field observations have yielded significant insight into the environmental and biotic controls on phytoplankton standing stock, community composition and rates of primary production. Riley [14] demonstrated phytoplankton biomass as a function of temperature, nutrients, zooplankton, and water depth. Despite using linear models with no interactions among predictors, he was able to explain 60–80% of the variance in the data, but his model coefficients were highly variable and difficult to interpret. Sverdrup [15] demonstrated the spring blooming of phytoplankton biomass in the Norwegian Sea as a function of the seasonal shoaling of the upper mixed layer increasing the average irradiance in a high-nutrient water column. Satellite observations and international sampling programs have significantly increased the temporal and spatial coverage of observations of upper ocean chlorophyll, sea surface temperature, and other environmental co-variables, allowing us to substantively determine how environmental conditions regulate phytoplankton biomass in the oceans. Analyses of remote-sensed chlorophyll document temporal shifts in the geographic distribution of chlorophyll biomass and total chlorophyll concentrations over the last 10 years [16]–[18] and the community compensation irradiance for the spring bloom in the North Atlantic [19]. Syntheses of satellite chlorophyll and field data indicate that climate change and/or climatic oscillations are responsible for changes in mean chlorophyll concentration and primary production over recent decades [16], [18], [20], [21]. In response to the need for predicted chlorophyll concentrations in modeled climate scenarios, Sarmiento et al. [22] predict annual mean log chlorophyll using a linear regression against temperature, salinity, length of the growing season, and the maximum winter mixed layer depth over 33 biogeochemical provinces. The coefficients in their linear models and their predictive power vary widely across regions.

We revisit the statistical idea proposed by Riley [14] using a flexible data mining technique to extract the environmental determinants of remote-sensed chlorophyll standing stock, incorporating variables that are mechanistic (light, nutrients, temperature, and mixed layer depth) and proxies for many unavailable data that vary regionally and temporally (location and month of year). Our model extends earlier efforts, permitting non-linear responses to environmental variables and allowing for interactions between nutrients and light [22]. This model can be used to determine which environmental conditions most strongly regulate photosynthetic standing-stock biomass, the details of the functional response for each environmental variable, and ultimately can be assembled to predict biomass and primary production under a climate change scenario in the context of a global circulation model.

## Materials and Methods

Our model requires large amounts of data spanning a large geographic region and many months. Satellite-based instruments provide such observations of phytoplankton chlorophyll, surface irradiance, and sea surface temperature. Other key variables cannot be observed from space but must be assembled from in situ observations and models to fill in gaps and are often available only as climatologies.

### Data

The assembly of global-scale databases of ocean color (chlorophyll, mg m^−3^) and environmental variables: photosynthetically available radiation at the sea surface (E, µmol photons m^−2^), macro-nutrient concentrations (NO_3_^−^, PO_4_^3−^, µmol L^−1^), mixed layer depth (MLD, m), and sea-surface temperature (SST, °C) provides us with a synoptic view of the distribution of phytoplankton and some of its environmental predictors. The geographic and temporal variables: latitude, longitude, and month of year are proxies for missing environmental variables that change with location and time. We mine these databases to extract the environmental controls and correlates of surface chlorophyll concentration, determining empirically how environmental conditions regulate chlorophyll biomass. Chlorophyll concentrations and sea-surface irradiance were obtained from the SeaWiFS project [23] and SST from the MODIS-Aqua project [24] and all were averaged to 1° resolution, monthly composites from 1999 to 2006. Nutrient climatologies (NO_3_^−^, PO_4_^3−^) were obtained from the World Ocean Atlas 2005 [25] and are defined on a 1° monthly grid. MLD fields are monthly climatologies using a temperature change of ±0.2°C defined on a 2° grid using NODC and NOAA data [26]. Data were restricted to lie within a box from 10°N to 60°N and 80°W to 0°, encompassing much of the North Atlantic, and totaling approximately 35,000 observations in each of 8 years. This region contains much of the range of variability in the variables seen on a global scale and is a well-studied and biogeochemically important part of the global ocean. The iron-limited regions in the Southern Ocean and Pacific were excluded because the iron data available are not of comparable quality to the macronutrients in the World Ocean Atlas. A subsequent analysis should expand the geographic extent, include iron as a predictor variable, and compare the model across different biogeographic provinces [27], [28].

### Analysis

Functional data analysis [29] extends linear and simple parametric models to permit responses depending on much more general functions. One approach to functional data analysis uses generalized additive models to estimate functions of predictor variables, adding together each effect to predict the response variable. This technique allows a modeler to extract relationships between the response and predictor variables from the data without making strong assumptions about the shape of the response function [30]. Using this approach, we model satellite-derived log chlorophyll concentration as a mean value plus the sum of several functions of environmental data. Our response functions are piecewise cubic polynomials depending on one or two predictor variables. To guard against over-fitting of the data, manifest by excessive oscillations in the estimated response functions, the likelihood function includes a penalty depending on the integral of the square of the second derivative of the response function. The data analysis was performed using R [31] and the generalized additive model tools developed by Wood [32], [33].

Our primary model incorporates the effects of light, nutrients, temperature, location and month of year as
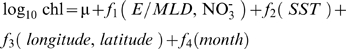
(1)where μ is the mean log chlorophyll and *f*_i_ are functions estimated from the data. We use log chlorophyll concentration as chlorophyll is approximately log-normally distributed. Sea-surface irradiance is divided by mixed layer depth to provide an estimate of the mean near-surface irradiance experienced by phytoplankton entrained in the mixed layer. A single function of mean irradiance and nitrate is used instead of two separate functions to permit the response to one factor to depend on the other. A natural hypothesis is that when one resource is limiting, there will be little or no effect on chlorophyll by changes in the other resources. The degree to which this is true can be deduced from the estimated *f*_1_. Geographic location is also allowed to affect chlorophyll through a single function of latitude and longitude, and the resulting function will be interpretable as a effect on chlorophyll determined by geographic location. These responses, plus responses to SST and month of year are all combined additively, and no interactions among these four responses are allowed in their effect on log chlorophyll. It is certainly possible that there are further interactions among these predictors. We chose to not include further interactions when obtaining the average effect of each combination of predictors in Eq. (1), because the data were insufficient (e.g., not all temperature and latitude combinations are available), and because visual interpretation of functions of three or more variables is difficult.

In addition to the functions in Eq. (1), we also estimated response functions predicting chlorophyll omitting each term in turn, and using formulations with only one response function at a time. Response functions were estimated using half of the data and the predictive skill was assessed using the other half of the data. The coefficient of determination (*r* ^2^ = 1−var(residual error)/var(data) ) describes the proportion of variation in log chlorophyll predicted by the model. The distribution of predicted log chlorophyll, a scatter plot of predicted vs. observed data, and the root mean square error was also used to assess the models.

## Results

**Figure 1:**
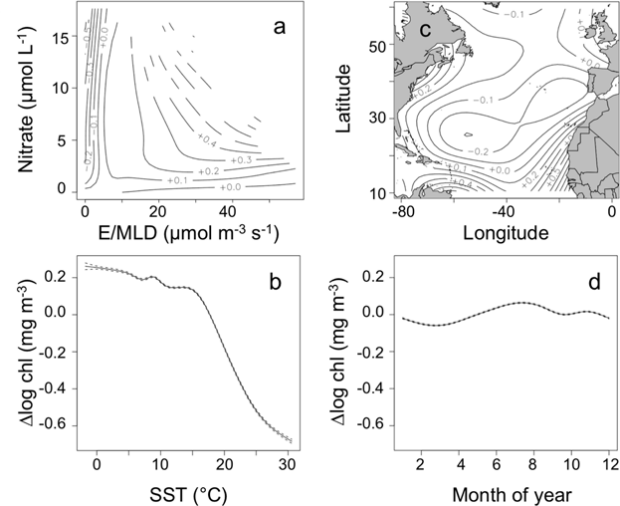
Functional response of log chlorophyll concentration (mg m^−3^) to 4 sets of predictors: (a) mean irradiance and climatological surface nitrate concentration, (b) sea surface temperature, (c) location in basin, and (d) month of year. Panels (a) and (c) are contour maps of two variable response functions. Dashed lines on panels (b) and (d) indicate point estimates of the standard error of the response function.

**Figure 2:**
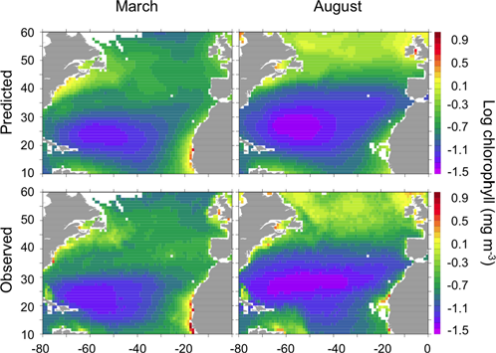
Log chlorophyll concentration, March and August 1999–2006 averages, predicted using Eq. (1) and observed satellite data.

Satellite-determined chlorophyll in the North Atlantic is approximately log normally distributed with a truncated left-hand tail. Chlorophyll concentration varies over approximately 3 orders of magnitude, ranging from 0.029 to 32.6 mg m^−3^, with a median of 0.17 mg chl m^−3^ (median log chlorophyll is −0.77). The standard deviation of log chlorophyll is 0.40, corresponding to a relative change in chlorophyll concentration of ±150%. A generalized additive model with functions (Eq. 1, in the methods) of SST, E/MLD and NO_3_^−^, latitude and longitude, and month of the year accounts for 83% of the variance in log chlorophyll (Figure 1). These functions show how log chlorophyll is affected by changing conditions; predicted log chlorophyll is computed as the mean −0.71 plus the deviations from mean log chlorophyll from each of the four functions in Fig. 1. Maps of predicted log chlorophyll replicate known geographic and temporal patterns in chlorophyll (Figure 2). The quality of the model can additionally be assessed with several other metrics: (i) there is a slight mismatch between predicted values and observed data (observed = 1.03 • predicted−0.015, *r* ^2^ = 0.83), (ii) the predicted variability in log chlorophyll (standard deviation = 0.36) is slightly less than for observed log chlorophyll (0.40) indicating a small amount of unpredicted variation, and (iii) predicted chlorophyll has a roughly 50% error (root mean squared error in log chlorophyll of 0.175), documenting a fair amount of residual variation, but not an excessive amount since satellite chlorophyll often has similar error compared to observed chlorophyll.

The influence of the individual predictors can be assessed by their relative contribution to the *r* ^2^ of a sequence of nested generalized additive models and their individual shape allows us to test inferences about mechanistic roles of each predictor. No single predictor could account for more that 51% of the variability in log chlorophyll and many predictors have substantially less influence (Table 1). Chlorophyll concentration is largely regulated by the availability of light, nutrients, and the effect of temperature, with half of the residual variability accounted for by factors depending on geographic location. Sea-surface temperature has a strong influence on log chlorophyll concentration, with the effects ranging from +0.2 to −0.6 log units relative to the mean. The log chlorophyll-SST response function (Figure 1b) has a fairly simple shape, indicating chlorophyll elevated above the mean for SST <15°C and rapidly decreasing as temperatures increase above that level. Used as a single predictor, SST can account for 51% of the variance in log chlorophyll, but when combined with mean irradiance and nitrate, increases the amount of variance explained from 47% (for mean irradiance and nitrate alone) to 65% (Table 1). Much of the variability in temperature is correlated with variation in light and nutrients, but there is some significant additional information in the SST record.

**Table 1:** Summary statistics for predictions of the full model (Eq. 1) and submodels: the proportion of variance in log chlorophyll concentration explained by the models (*r* ^2^) and the root-mean-square deviation of predicted from observed log chlorophyll (RMS error).

Model Predictors	*r* ^2^	RMS Error
E/MLD & NO_3_^−^, SST, Lat & Long, Month	0.83	0.17
E/MLD & NO_3_^−^, SST, Lat & Long	0.83	0.17
E/MLD & NO_3_^−^, SST	0.65	0.24
E/MLD & PO_4_^3−^	0.56	0.27
E/MLD & NO_3_^−^	0.47	0.29
SST	0.51	0.28
PO_4_^3−^	0.49	0.29
NO_3_^−^	0.39	0.31
E/MLD	0.04	0.39
Month	0.02	0.40

As the primary resources necessary for growth, light and nutrients might be expected to be important predictors of biomass and the response function should document the need for both resources to sustain higher concentrations of biomass. Since other factors (e.g., grazing) also regulate biomass, the response function for light and nutrients may not be exactly what would be expected from a physiological point of view. Contours of the response functions indicate how light (E/MLD) and nutrients (nitrate) account for deviations from mean log chlorophyll (Figure 1a). The combined light-nutrient function closely approximates a minimum function, exhibiting characteristic ‘L’ shaped contour lines. At high light, but low nutrients, changes in light lead to relatively small changes in log chlorophyll, while changes in nutrients lead to much larger changes in log chlorophyll; for example the 0.3 contour on the right half of Fig. 1a is nearly parallel to the E/MLD axis. Using phosphate instead of nitrate produced similar but less consistent ‘L’ shaped contour lines, and in the full model the amount of variance explained was unchanged.

Geographic information accounts for an increase in the explained variance in log chlorophyll from 65 to 83% and month of year adds <1% to the *r* ^2^ of the full model. The latitude-longitude response shows some increases in chlorophyll near coasts and areas of known upwelling as well as a minor latitudinal gradient, increasing away from the mid-latitudes (Figure 1c). A function of longitude and latitude appears to act as a catch-all proxy for many additional factors which affect chlorophyll concentrations and vary spatially such as periodic nutrient inputs and micronutrient availability. The final predictor in the model accounts for variation in chlorophyll due to time of year. We find that the spring and fall blooms do not appear in this function (Figure 1d) having been accounted for by light and nutrients and that the purely temporal response function has a small amplitude (±0.06 log units, corresponding to ±15% variation in chlorophyll concentration).

## Discussion

Climate change is altering the temperature and pH of the oceans [34], species phenology [35], [36], and the size of the major ocean gyres [16]. Ocean models predict future changes in large-scale circulation, currents, patterns of stratification, and thus the distribution of nutrients in the euphotic zone and the relative depth of the mixed layer and compensation depth. Our model provides predictions of phytoplankton chlorophyll biomass based on environmental parameters that can be incorporated into models of future ocean environments. Predicted chlorophyll biomass can then be used to estimate rates of primary and export production [1], [18], [37]. The empirical functions we obtain from functional data analysis are interpreted below as ecological responses of the phytoplankton community, incorporating growth and loss terms (e.g., grazing). These responses are not purely physiological but are synthetic combinations of many factors.

The model predicts 83% of the month-to-month variation in log chlorophyll in the North Atlantic over a span of 8 years. Light and nutrient resources available to phytoplankton account for the majority of this variation, indicating that resources determining biomass-normalized growth rates can be used to predict standing stock chlorophyll biomass. This is not necessarily expected as chlorophyll concentration is a pool and not a rate. Standing stock is affected by the balance between factors responsible for growth and loss; changes in standing stock are due to transient imbalances between growth and loss. Environmental factors that promote growth appear prominently in our model, but loss terms such as grazing by zooplankton, aggregation and sinking, advection, and cell death by viruses, parasites, or apoptotic mechanisms are not explicitly included. The fact that we can predict chlorophyll concentration from light and nutrients, which determine growth rates, leads us to conclude that loss rates are often dependent on growth rates or chlorophyll concentration and that biomass is effectively regulated by bottom-up factors.

The availability of light and macronutrients limit phytoplankton growth in much of the ocean [38]–[41]. If biomass is regulated by availability of resources through a mechanistic link to growth rates then this should be identifiable in the response function for light and macronutrients. As predicted the effect of average irradiance and nitrate concentration on chlorophyll concentration closely resembles a minimum function (Figure 1a), showing that in general either light or nitrate limits the concentration of chlorophyll biomass. This is precisely the effect resource availability should have on growth rate, indicating that the bottom-up effects of resources on growth rates have a dominant role in regulating biomass. Our result is consistent with the common observation that nitrate limits biomass in the majority of the North Atlantic in the summer. There are minor deviations from the minimum function where neither light nor nitrate is strongly limiting; these deviations are most common at low E/MLD and low nitrate concentrations. At low nitrate concentrations an increase in irradiance has less of an effect on chlorophyll than at higher nitrate concentrations. Co-limitation can be identified along a vector through the bend in the L-shaped contours of the minimum function (Figure 1a). Phosphate is limiting in parts of the subtropical North Atlantic [41] and our nitrate-based model predicts higher than observed chlorophyll in this region. If mean irradiance and phosphate concentration are used in Eq (1), the predictive power of the full model is essentially unchanged, but the response function (not shown) deviates more from a minimum function because phosphate is not limiting in most of the North Atlantic. Similar over-predictions of chlorophyll will be observed in Fe-limited regions; models outside the North Atlantic should incorporate both Fe and phosphate.

In our framework, SST is the best single predictor of log chlorophyll, explaining roughly half of the total variability (Table 1). Sea-surface temperature affects photosynthetic rates [2], [42], is correlated with MLD, and can be linked to nutrient availability [43], [44] and temporal changes in irradiance, nutrients, and stratification [45]. The temperature response function is very different from the physiological relationship between growth rate and temperature, which generally increases exponentially until a viability threshold is exceeded [42]. Each phytoplankton species has an optimal temperature for growth, but the global community contains sufficient diversity that temperature has little direct effect on regulating biomass through direct physiological mechanisms. A potential interpretation of the response (Fig. 1b) is that it is the signal of a temperature-nutrient relationship: surface nutrients decline with increasing temperature above ∼15°C, although the details of this relationship vary with latitude [44]. If nutrients are not included in the model and SST is used as a single predictor, the magnitude of the temperature effect is greatly increased (results not shown), indicating that much of the effect of nutrients in the temperature data is, in fact, represented by the nutrient data. To the extent that nutrients and SST are correlated, the statistical model is unable to distinguish the effects of one predictor from the other; the chlorophyll response is divided between the two predictors. If dramatic changes in climate occur, perhaps due to a regime shift, leading to changes in these correlations, the model predictions may be in error, although the approach taken here is conservative because current responses are divided among the correlated predictors.

An alternate interpretation for the decrease in chlorophyll described by the SST response function is as a change in the balance between phytoplankton growth and losses by grazing. The growth rate of herbivorous protists and copepods increases more rapidly than the growth rate of phytoplankton as temperature increases [46]. The temperature response function (Fig 1b) may be a signal of increased grazing pressure, and a change in the relative effect of growth and loss terms on biomass, with the exponential decrease in chlorophyll biomass above 15°C caused by the exponential increase in growth of predators relative to prey. Differential responses of organisms from different trophic levels to changes in climate (and associated environmental variables) may have drastic and very difficult to predict effects on marine food webs [35].

Light, nutrients, and temperature are primary determinants of phytoplankton growth rate and biomass, but many additional physical and chemical factors influence chlorophyll concentration and have not been included in the model. Geographic location is correlated with many factors influencing average chlorophyll, including bathymetric effects on mixing and advection and resource input from aeolian and riverine sources. Variability on scales smaller than our sampling resolution (1°×1° and 1 month) is hidden in our analysis and could bias the relationships between mean environmental conditions and predicted chlorophyll concentration, because phytoplankton respond not only to average resource levels, but also to the amplitude and frequency of variability in irradiance and nutrients that changes with vertical stratification [47]. Geographically localized sources of variation in resources arise from several sources including episodic inputs of nutrients from rivers, mixing due to storms and eddies, and vertical mixing at the Brunt-Väisälä frequency [48], [49]. The contours of the latitude-longitude response function show a broad latitudinal trend in log chlorophyll, with the largest increases near coastlines (Fig 1c), showing that location acts as an effective proxy for many factors beyond light, nutrients, and temperature. An ideal model would not use latitude and longitude explicitly, but this term in the model is a convenient short-hand given the complexity of the problem and limitations in some of the available data.

Our model is memoryless, meaning that neither the history of chlorophyll concentration nor our predictor variables is used in the prediction of chlorophyll. Phytoplankton grow rapidly, with a potential of 30 or more doublings per month, and factors other than seed population size must dominate the regulation of chlorophyll concentration, or we should observe changes on the order of a factor of 10^9^ (∼2^30^) during a month. The availability of light and nutrients, temperature, and geographic location largely account for our sense of temporal sequence in the distribution of chlorophyll. To test this idea, we added the month of the year to our generalized additive model to see how much residual variation could be explained. The resulting function (Figure 1d) has a small amplitude, ±0.06 log units, with a peak in July and trough in March and added about 1% to the *r* ^2^ of the model (Table 1). The North Atlantic spring bloom does not appear in this function as it has already been accounted for by light and nutrients. These small corrections to the model with three response functions (*f*_1_, *f*_2_, *f*_3_) indicate that month of year is a minor predictor after other factors are incorporated. If month of year is used as the sole predictor (not shown), a stronger seasonal trend with troughs in January and August, and a peak in April is obtained, but only accounts for 3% of the total variation in log chlorophyll.

Our approach demonstrates that a fairly simple statistical model can account for the majority (83%) of variation in log chlorophyll concentration across 8 years of North Atlantic data. Bottom-up factors (mean irradiance and nitrate) alone account for 47% of the variation and demonstrate a mechanistic relationship: biomass is affected by changes in light or macronutrient, whichever is limiting. Further study will refine this approach, perhaps by geographic sub-division, e.g., into biogeographic provinces [27], but the results from this attempt combining satellite data and functional data analysis shows promising results. Anthropogenic climate change is expected to change many of the environmental variables that regulate the distribution of chlorophyll biomass, most notably temperature, mean irradiance, and nutrient availability. The simplicity of the model suggests a computationally simple way to predict changes in chlorophyll distribution with changes in mean irradiance, nutrient concentrations, and temperature, which can be predicted by ocean general circulation models.
